# Adipose Tissue in Chagas Disease: A Neglected Component of Pathogenesis

**DOI:** 10.3390/pathogens14040339

**Published:** 2025-03-31

**Authors:** Vitória França dos Santos Pessoa, Mariana Hecht, Nadjar Nitz, Luciana Hagström

**Affiliations:** 1 Interdisciplinary Laboratory of Biosciences, Faculty of Medicine, University of Brasilia, Brasília 70910-900, Brazil; vitoria.pessoa@aluno.unb.br (V.F.d.S.P.); marianahecht@gmail.com (M.H.); nadjarnitz@gmail.com (N.N.); 2 Faculty of Physical Education, University of Brasília, Brasília 70910-900, Brazil

**Keywords:** *T. cruzi*, adipocytes, high-fat diet, pathophysiology

## Abstract

Chagas disease (CD), caused by the protozoan *T. cruzi*, is a serious public health issue with high morbidity and mortality rates. Approximately 7 million people are infected, mostly in Latin America. The pathogenesis is multifactorial and poorly elucidated, particularly regarding the role of adipose tissue (AT). This review aims to explore the complex relationship between *T. cruzi* and AT, focusing on the possible role of this tissue in CD, as well as to explore the impact of diet on the progression of the disease. *T. cruzi* infects adipocytes, affecting their function. Chronic infection alters adipose physiology, contributing to systemic inflammation and metabolic disturbances. Adipokines are dysregulated, while markers of inflammation and oxidative stress increase within AT during CD. Additionally, the immune response and clinical aspects of CD may be influenced by the host’s diet. High-fat diets (HFDs) impact parasite burden and cardiac pathology in murine models. The complex interaction among *T. cruzi* infection, AT dysfunction, and dietary factors underscore the complexity of CD pathogenesis. Despite accumulating evidence suggesting the role of AT in CD, further research is needed to elucidate its clinical implications. Understanding the bidirectional relationship between AT and *T. cruzi* infection may offer insights into disease progression and potential therapeutic targets, highlighting the importance of considering adipose physiology in CD management strategies.

## 1. Introduction

Although Chagas disease (CD) is endemic in Latin America, it affects about 6 to 7 million individuals worldwide. Due to the migration of infected people, this neglected tropical disease is found, for example, in the United States of America, as well as in several countries in Europe and Africa [1]. CD is caused by the flagellated protozoan *T. cruzi*, an obligate intracellular parasite known for its ability to persist in mammalian hosts indefinitely after the initial infection [2,3]. Thus, CD is a life-threatening illness with distinct acute and chronic phases. Most acute infections are asymptomatic or present nonspecific symptoms. During this phase, which lasts 4 to 8 weeks post-infection, parasitemia is elevated [2,4].

Conversely, in the chronic phase, parasitemia is absent or very low. Most patients will remain asymptomatic for life. However, approximately 30% of infected people will develop cardiac and/or gastrointestinal symptoms around 30 years after the initial infection [2,4]. The drugs currently available for the treatment of CD are effective in the acute phase but are not satisfactory in the chronic phase [5].

**Figure 1:**
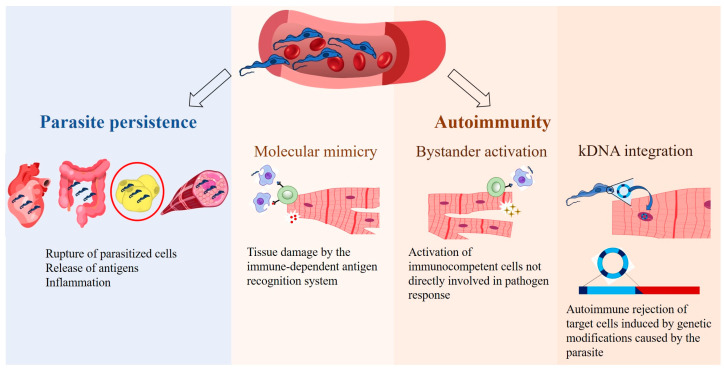
Main theories on the pathogenesis of Chagas disease. According to the parasite persistence theory, *T. cruzi* replicates within host cells, causing the rupture of infected cells and releasing antigens that trigger the host’s immune response, leading to inflammation. The autoimmune theory suggests that the immune system targets the host’s own tissues, causing chronic inflammation through the following mechanisms: molecular mimicry, bystander activation, and potentially *T. cruzi* kinetoplast DNA (kDNA) integration. Erythrocytes (https://beta.scidraw.io/drawing/634), heart (https://beta.scidraw.io/drawing/277), intestine (https://beta.scidraw.io/drawing/296), adipocyte (https://doi.org/10.5281/zenodo.3926133), myocyte (https://doi.org/10.5281/zenodo.3926129), and macrophage (https://beta.scidraw.io/drawing/221) were obtained from https://scidraw.io/. Accessed on 19 February 2025.

This gap in effective chronic-phase treatments underscores the need for a deeper understanding of the disease’s progression. Despite decades of research, the pathogenesis of CD is still debated and questioned, leading to the development of theories aimed at elucidating the mechanisms underlying the characteristic symptoms of its late phase. In fact, CD pathogenesis is multifactorial and very complex, influenced by various factors such as parasite strain, the persistence of *T. cruzi* in different host tissues, and the host’s metabolic and immunological status (for review, see [6]). Thus, two primary theories have emerged: parasite persistence and autoimmunity mechanisms [4] (Figure 1).

The first theory proposes that tissue damage initiates with parasite infection. *T. cruzi* demonstrates wide infective versatility, invading diverse tissues and cell types [2,7], including macrophages, skeletal and cardiac muscle cells, epithelial cells, and adipocytes [8]. Actively proliferating within cells, the parasite causes their mechanical rupture, leading to microvascular spacing, myocytolysis, and alterations in enteric and cardiac nerves [7] (Figure 1). Importantly, *T. cruzi* can evade the immune system, enabling its persistence throughout the host’s lifespan [9,10,11]. Recent studies corroborate the role of parasite persistence in the pathophysiology of CD [12,13,14].

Numerous findings on the tissue distribution of *T. cruzi* have further supported the theory of parasite persistence [9,13,15,16]. Quantitative PCR (qPCR) and bioluminescence imaging systems have demonstrated that *T. cruzi* circulation is highly dynamic, showing a tropism for the gastrointestinal tract and adipose tissue (AT), among other tissues (for review, see [10]). These tissues may act as reservoirs for the parasite in the chronic phase of the disease and facilitate its eventual migration to the myocardium, resulting from years of myocarditis and cardiac fibrosis [17,18]. Amastigotes of *T. cruzi* were observed in cultures of adipocytes differentiated from human AT-derived stem cells, reinforcing the idea that AT also serves as a reservoir for the parasite in humans [19]. However, the association between the presence of *T. cruzi* in AT and CD symptoms remains inconclusive. The need for ultrasensitive techniques to detect the parasite in injured areas suggests that *T. cruzi* alone is not the single cause of tissue injury, indicating a potential damage amplification mechanism [20].

In this context, even though it remains controversial, autoimmunity has been proposed as a mechanism triggered by *T. cruzi* in the host. This self-reactive hypothesis proposes that, regardless of the initial cause, a powerful immunological stimulus disrupts the body’s surveillance mechanism, playing a critical role in cardiac tissue damage [6,21]. This hypothesis was initially demonstrated in vitro by Santos-Buch and Teixeira [22], who observed the destruction of cardiac fibers in rabbit fetuses by lymphocytes derived from chronic chagasic rabbits, whereas control rabbit lymphocytes did not attack cardiac cells [22]. Thus, it was found that immunocompetent lymphocytes from chagasic rabbits could destroy myofibers and neurons of the parasympathetic nervous system in healthy, uninfected rabbits [23].

Several mechanisms have been proposed to explain autoimmunity, including the cross-recognition of similar antigens between the parasite and host tissues (molecular mimicry), activation of immunocompetent cells not directly involved in pathogen response (bystander activation), and potentially the genetic material exchange between the parasite and host (integration of minicircles of *T. cruzi* kinetoplast DNA [kDNA]) [6,21,23] (Figure 1).

Although distinct, these theories (parasite persistence and autoimmunity) are not mutually exclusive. It is highly probable that CD results from complex interactions between the parasite, the host’s immune system, and other genetic factors [8,24].

In this context, AT emerges as an additional element in this intricate chain, contributing to the persistence of the parasite by acting as a reservoir [25,26,27] and providing an environment rich in inflammatory mediators [27], which may aid in the development of chronic Chagas cardiomyopathy (CCC). Thus, this review will explore the known mechanisms by which AT influences CD pathogenesis, including its role in immune modulation, metabolic alterations, and parasite–host interactions. Additionally, this review will investigate the impact of diet on the physiology of AT and CD. We will primarily focus on white adipose tissue (WAT), as the involvement of brown or beige AT in the context of CD remains underexplored.

Of note, due to *T. cruzi*’s ability to invade a wide range of cells and tissues, Añez and Crisante [28] propose that the parasite has no preference, suggesting that specific tropism does not exist [28]. Nevertheless, most studies discuss the parasite’s preference for infecting certain tissues over others (due to different factors, e.g., the strain) and, therefore, use the concept of tissue tropism.

It is also important to highlight that only a few research groups study the role of AT in the context of *T. cruzi* infection, and most experiments have been conducted in animal models. Although the results from murine models cannot be directly translated to human CD, they remain crucial for understanding the disease. Actually, studies in humans are scarce. However, research on differentiated human adipose cells showed that *T. cruzi* parasites these cells [19,29,30]. Similarly, in chronically infected patients, *T. cruzi* DNA was detected in AT [26].

## 2. Complexity of Adipose Tissue: Structure and Function

AT is an intricate multicellular organ exhibiting significant biological variability depending on the depot level, anatomical location, and metabolic state. It exerts extensive physiological actions influencing processes such as immunity and inflammation (for review, see [31]). Actually, beyond the adipocytes, many cell types are present, interacting spatially and functionally [32], including vascular components, nerve tissue, immune cells, endothelial cells, fibroblasts, and other adipocyte precursors [33,34]. Immune cells (e.g., macrophages, neutrophils, eosinophils, dendritic cells, and lymphocytes) are an important part of AT that secrete various pro-inflammatory cytokines that can modulate the physiological state of the organism (for review, see [35]). Indeed, due to the abundance of immune cells within AT, adipogenic cells, and their precursors, this tissue may play a role in combating infections [33].

**Figure 2:**
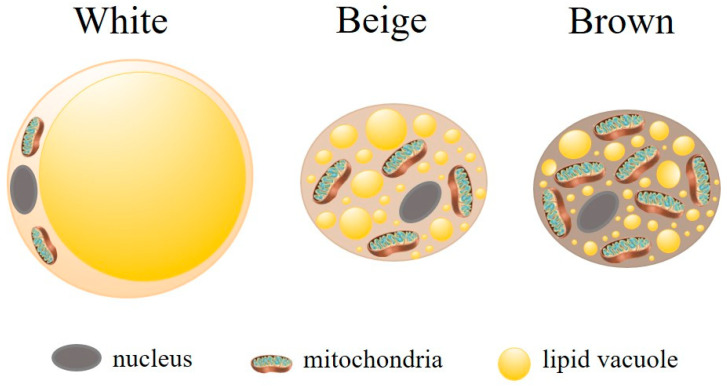
Main types of adipocytes. There are three main types of adipocytes, each differing functionally and morphologically. White adipocytes are large and contain a single lipid droplet and few mitochondria. Their primary function is energy storage. Brown adipocytes have numerous small lipid droplets and a high number of mitochondria. Their main function is thermogenesis. Beige (or brite) adipocytes display intermediary characteristics, highlighting the remarkable plasticity of adipose tissue. Adipocyte (https://doi.org/10.5281/zenodo.3926133) and mitochondria (https://doi.org/10.5281/zenodo.7590755) were obtained from https://scidraw.io/. Accessed on 19 February 2025.

WAT and brown adipose tissue (BAT) are the main types of AT, currently recognized as an endocrine organ with distinct functions [34]. WAT is the predominant type in human adults [36] and is mainly involved in lipid storage and release to maintain energy homeostasis. Adipocytes, the principal cell type of WAT, are characterized by a large, unilocular lipid vacuole in the cytoplasm, which facilitates substantial lipid storage (for review, see [31,37]), while containing few mitochondria [38] (Figure 2). Beyond their role in energy storage, white adipocytes in WAT secrete various bioactive substances, including peptides (e.g., adipokines) [38], proteins [39], and microRNAs (miRNAs) [40], which influence systemic metabolism and modulate the immune response. WAT plays a critical role in energy balance, insulin sensitivity, and inflammation. In particular, it secretes pro-inflammatory adipokines, such as tumor necrosis factor-alpha (TNF-α) and interleukin-6 (IL-6), which influence the immune system and contribute to inflammatory processes [41]. These cells also serve as a reservoir for immune cell infiltration, as almost all types of innate and adaptive immune cells are found in WAT, further underscoring their involvement in both metabolic and immune regulation [41].

Although BAT is found in human adults in small depots localized in specific areas (neck, supraclavicular, axillary, paravertebral, mediastinal, and epigastric regions), it is mainly present in small mammals (e.g., mice) and human newborns (for review, see [37]). BAT is more metabolically active than WAT, playing a role in thermogenesis (non-shivering heat generation and dissipation of energy), weight loss, and the secretion of adipokines [31,32,42]. Unlike WAT, brown adipocytes contain several cytoplasmic lipid droplets of different sizes and numerous mitochondria (Figure 2). The high iron and cytochrome content resulting from the dense mitochondrial population, along with extensive vascularization, imparts the characteristic brown coloration to BAT [43].

Another type of AT is the beige or brite (brown-like in white) adipocytes. These cells emerge in WAT depots in response to external stimuli, such as prolonged cold exposure in response to sympathetic activation [31,43]. Beige adipocytes contain multilocular lipid vacuoles and abundant mitochondria (Figure 2). Functionally, beige adipocytes resemble BAT expressing brown adipocyte genes (for review, see [44]). Their role in thermogenesis and the regulation of insulin resistance and obesity has been studied. Beige adipocytes also possess a secretory capacity that exerts endocrine and paracrine effects (for review, see [31]).

During gestation and lactation, subcutaneous WAT transdifferentiates into pink AT (PAT), a mammary glandular tissue that acquires a pink color and is capable of producing and secreting milk. PAT contains multiple cytoplasmic lipid droplets, an apical surface with microvilli, a large and round nucleus, a well-developed endoplasmatic reticulum, a Golgi complex, and granules containing milk (for review, see [44]). The conversion of WAT to BAT or beige AT, as well as the WAT to PAT, is reversible, reinforcing the enormous plasticity of AT [45].

This review will primarily focus on WAT, as the involvement of BAT or beige AT in the context of CD remains underexplored.

## 3. Pathogens in White Adipose Tissue

Various infectious agents accumulate at different sites within the AT, a tissue with great longevity that is rich in nutrients. These characteristics facilitate the persistence of pathogens within the host during chronic infections [18,41]. Noteworthily, the immune response to the presence of foreign microorganisms varies depending on the site within the AT where they are localized, such as the cytoplasm of adipocytes, interstitial spaces, inside vessels, immune cells, and stromal vascular fraction [18]. Additionally, AT interacts with cells from other tissues through the release of adipokines and cytokines, which probably influence the progression of diseases [37,41].

The relationship between microorganisms, host AT, variations in host fat accumulation, and pathogenesis of infections is complex and not yet fully elucidated. Indeed, microbes can disrupt the normal functioning of AT [18], and the accumulation or decrease of fat in different organs can impair their normal functions. The dysregulation of AT can lead to systemic impairments, affecting other physiological systems [46].

Various pathogens persist in AT during infection, such as *Mycobacterium tuberculosis* [47,48], *Plasmodium berghei* [49,50], SARS-CoV-2 [41,51], and *Trypanosoma brucei* [52,53,54]. *Leishmania infantum* was also observed by immunolabeling at 40 weeks post-infection in WAT, as well as in BAT. Furthermore, it has been demonstrated that *L. infantum,* present in AT, maintains its infectivity [55]. Barthelemy et al. [41] provide a list of pathogens (bacteria, viruses, and parasites) documented to infect AT and eventually persist within them [41].

*T. brucei* and *T. congolense* were found in different AT deposits in naturally infected livestock animals. Subcutaneous WAT and gonadal WAT exhibited the highest rates of trypanosome detection [55]. This finding reinforces the role of AT as reservoirs for trypanosomes not only in mice [56] but also in natural hosts (cows, goats, and sheep) [52]. Moreover, it has been proposed that *T. brucei* within AT exhibits resistance to pharmacological treatment [54], underscoring the necessity to further elucidate the interactions among parasites, AT, and the host.

In the case of *T. cruzi*, AT serves as an early target for the parasite and acts as a reservoir during chronic infection [7,25,57]. The bidirectional interaction between the AT and *T. cruzi* may imply two distinct consequences. First, since *T. cruzi* resides in the AT, it benefits from the long half-life of adipocytes in both humans and mice, which provides the parasites with easy access to nutrients, including lipids and fatty acids [18]. Both are essential for the growth and survival of *T. cruzi* [58]. This may allow the parasites to remain in a quiescent state [18], evading the host’s immune response [59]. Indirectly, the specific location of *T. cruzi* in AT might protect the heart. On the other hand, even though the dormant forms of *T. cruzi* in AT may have a minor role in disease progression, they sustain the parasite persistence in the host [10,60], leading to an inflammatory response that would contribute to tissue damage, potentially reactivating the disease [25].

Amastigotes, the intracellular replicative form of *T. cruzi*, are found in the cytoplasm [3]. Thus, in WAT, they are close to lipid droplets [18]. Given that trypanosomes exhibit limited fatty acid oxidation during their life cycle, it was suggested that they may rely on local lipolysis to satisfy their energy requirements [58] and sustain these replicative stages.

## 4. Consequences of *T. cruzi* Presence in Adipose Tissue

Numerous studies underscore the importance of AT as a critical target for *T. cruzi* infection [25,61,62]. Indeed, the presence of the parasite in fat cells alters the function of AT, leading to local and systemic inflammation, metabolic abnormalities, and changes in cardiac physiology, thereby aggravating CD [17,60,63,64,65]. The involvement of epicardial and pericardial AT in CCC has also been suggested [65].

Several mechanisms may be involved, including adipocyte apoptosis or enhanced adipogenesis [64], mitochondrial dysfunction [63,65], oxidative stress [65,66,67,68], and endoplasmic reticulum stress in the myocardium [68]. Additionally, infection-specific extracellular vesicles (adiposomes) released by AT have been shown to modulate immune and metabolic gene expression in various cell types, such as cardiomyocytes and macrophages [65]. Undeniably, various pathways are altered during acute infection and continue to exhibit changes throughout the chronic phase [60], facilitating the parasite’s persistence within the host [65].

The presence of *T. cruzi* within AT has been demonstrated both in cell culture [19,25,30,69] and in murine models [70,71,72,73,74,75]. Bioluminescence imaging, which enables the visualization of parasite-retaining regions, confirms the attraction of *T. cruzi* to AT [9,76] with implications for the progression of CD. In mice, it was observed that cardiac parasitism was lower compared to the WAT and BAT at 15 days post-infection (dpi) [25,57]. Likewise, Zaki et al. [75] reported a higher parasite burden in epididymal fat than in the heart during the early stages of infection [75]. This preferential tropism of *T. cruzi* for AT in the acute phase may explain the relatively reduced cardiac damage observed in this period [17].

In the acute phase of *T. cruzi* infection, significant alterations are observed in the transcriptional program of adipocytes [25]. Consequently, this leads to pro/anti-inflammatory imbalance in AT and impaired lipolysis [77].

The immune function of AT can be modulated by *T. cruzi* infection [27]. Fifteen/seventeen days post-infection with *T. cruzi*, mice exhibit macrophage infiltration into AT [27,57,78]. The interaction between macrophages and AT results in persistent inflammation [79]. This phenomenon remains in the chronic phase, concomitant with the persistence of parasites contributing to the pathophysiology of CD [27]. Additionally, infected animals showed a decrease in body fat, which may also contribute to the pathogenesis of CCC [25,78]. Many factors can be responsible for the atrophy and loss of adipocytes, such as the inability of adipocytes to store fatty acids and the lysis caused by parasites [78]. The reduction in adipocytes allows the translocation of parasites to other tissues and may trigger pro-inflammatory responses with immune cell infiltration, potentially leading to the reactivation of the infection [25].

It was also suggested that inflammatory factors released into the bloodstream during infection may participate [30,78]. In this context, the supernatant of the indirect cultivation between AT differentiated from human AT-derived stem cells subsequently infected by *T. cruzi* and human peripheral blood mononuclear cells (PBMCs) indicated enhanced levels of pro-inflammatory cytokine interleukin-6 (IL-6) and a decrease in tumor necrosis factor-alpha (TNF-α) [30].

In mice, an upregulation of inflammatory markers in AT during acute *T. cruzi* infection was observed [25,53,78,80], including IL-6 and TNF-α [25,78]. In chronic chagasic patients (n = 190), IL-6 levels were elevated compared to the control group, while TNF-α levels showed only an upward trend [81].

Through the secretion of multiple adipokines, the AT controls the metabolism of various organs, such as the heart. Thus, AT dysfunction in disease states may contribute to adverse clinical outcomes (for review, see [82]). Recognizing that adipose tissue modulates the metabolism of various organs, including the heart, through the secretion of multiple adipokines, numerous researchers have investigated its role in the pathogenesis of CD. The inflammatory phenotype in mice in the early phase of *T. cruzi* infection was associated with a decrease in peroxisome proliferator-activated receptor gamma (PPAR-γ), adiponectin (AD), and leptin [78]. The transcription factor PPAR-γ is a regulator of adipogenesis [77]. AD, an adipocyte hormone, has anti-inflammatory and cardioprotective actions [82] and plays critical roles in energy regulation, glucose, and lipid metabolism (for review, see [83]). Leptin, primarily produced by adipocytes and released in proportion to body fat deposits, is a pro-inflammatory adipokine and a key mediator in the pathophysiology of cardiovascular disease, being involved in vascular remodeling, oxidative stress, and hypertrophy in the heart (for review, see [84]).

PPAR-γ has been observed to be suppressed during *T. cruzi* infection [77]. Conversely, Nagajyothi et al. [57] demonstrated differential responses of BAT and WAT to acute *T. cruzi* infection, with a reduction in PPAR-γ expression and an upregulation of TNF-α in BAT, whereas PPAR-γ was elevated in WAT. These contrasting responses may be attributed to variations in parasite load between the two types of adipocytes [57]. Additionally, the activating transcription factor-7 (ATF-7), a protein required for adipocyte differentiation, showed no changes during *T. cruzi* infection [77].

Despite the anti-inflammatory and cardioprotective properties [82], the role of AD in cardiovascular disease remains controversial, as well as in Chagas cardiomyopathy. Due to its anti-inflammatory and cardioprotective properties [82], AD has been widely investigated in Chagas cardiomyopathy. However, its role in CD and other cardiovascular disorders remains controversial. Some authors have reported that AD levels are reduced during acute *T. cruzi* infection in both in vitro [69] and in vivo [25,27,57,77] studies. Indeed, Santamaría et al. [77] observed lower AD concentrations in murine plasma and a lack of AD protein expression in WAT in the acute phase of CD [77]. Combs et al. [25] showed in mice that AD plasma levels were reduced at 30 dpi compared to controls; however, at 60 and 90 dpi, the values were similar [25].

On the other hand, other studies stated that AD overexpression can occur in chronic *T. cruzi* infection [64,65], potentially resulting in adverse effects. In two different studies with 15 patients with CCC, the amount of serum AD was significantly higher [85] or tended to increase compared to controls [86]. The reduced fat mass in the group with CCC could explain these findings [85,86]. In fact, in the context of fat loss, adipocytes may fail to express adequate levels of AD [65].

Leptin plays a role in innate and adaptative immunity [41]. Fernandes et al. [84] demonstrated that serum leptin levels were reduced in patients with CD (indeterminate chronic phase, with electrocardiogram abnormalities and heart failure) compared to healthy individuals [84]. More recently, Barbosa-Ferreira et al. [85] and Dabarian et al. [86] found no differences in leptin and AD levels between individuals with CCC and the control group. In these studies, body mass index (BMI) was similar between groups [85,86]. In contrast, when the BMI was higher in patients chronically infected than in controls, leptin levels were increased, and AD tended to decrease [81].

In a study involving the ablation of fat cells in mice during the indeterminate form of the chronic phase (90 dpi), Lizardo et al. [64] demonstrated an increase in cardiac pathology, reinforcing the idea that adipocytes contribute to the progression of CCC. In the hearts of these mice, there was a higher immune cell infiltration, more pronounced histological changes, and an increased number of parasites compared to infected mice without fat cell ablation. Several signaling pathways might be implicated [64]. Furthermore, the parasite persistence in AT in chronically infected mice was shown, suggesting that the presence of *T. cruzi* and the associated inflammatory infiltrates alter AT physiology (i.e., imbalance between adipogenesis/lipolysis) [64].

Markers of oxidative stress that have been associated with heart disease show increased levels of both WAT and BAT in CD. Simultaneously, the antioxidant response in these tissues is impaired in *T. cruzi*-infected mice. Thus, CCC has been associated with a high degree of inflammation and oxidative stress within AT [67].

While several studies have documented the presence of *T. cruzi* in WAT and/or BAT [25,26,87,88], the distinct consequences of parasitism in each tissue type, mainly in BAT, have received limited attention. Given their distinct cellular, molecular, and physiological characteristics, including antagonistic functions [41], the adipocyte response infection on WAT and BAT is probably different [89].

In view of the above, the presence of *T. cruzi* in AT plays a critical role in the progression of CD, influencing various pathological processes such as inflammation, metabolic disturbances, and immune dysregulation. The parasite’s preference for AT, particularly in the acute phase, leads to alterations in adipocyte function, including apoptosis, impaired lipolysis, and changes in adipokine secretion. These disturbances contribute to the persistence of *T. cruzi* and exacerbate cardiac pathology during the chronic phase of the disease.

## 5. Influence of a High-Fat Diet on the Physiology of Adipose Tissue and Chagas Disease

Human health is closely linked to dietary habits. Most of the global population follows Western diets, characterized by high calorie and fat intake. This dietary pattern is becoming prevalent in Latin America, an endemic region for CD [90], often leading to overweight and obesity, which may have effects on the interaction between *T. cruzi* and the human host [17]. Indeed, more than half of the female population in Latin America is either overweight or obese [91].

A study involving 66 patients with chronic CD in Brazil found that 83% exhibited a sedentary lifestyle and 94% were overweight or obese [92]. In Argentina, 42% of 190 *T. cruzi*-chronically infected individuals had BMI values compatible with obesity [81]. Despite these findings, there is still no consensus that *T. cruzi* infection causes obesity, that obese individuals are more susceptible to poor prognosis of CD, or that the parasite load is higher in overweight individuals [7,25,26]. However, it is established that the high-fat diet (HFD) has metabolic consequences and induces a pro-inflammatory state in AT [75]. The question that remains unresolved is whether an HFD improves or exacerbates CD.

Of note, Janoschek et al. [93] emphasize that a wide variety of diets are used in murine studies. The nutritional composition is quite variable, underscoring the lack of standardization. The most commonly used HFD in studies over the past decade provides 60% of its calories from fat. The studies selected for this review, which allow an analysis of the influence of HFD on AT and CD, have employed this diet, except for the study by Zaki et al. [75], where the HFD contained 20% fat, 20% protein, and 48% carbohydrate [94].

Without *T. cruzi* infection, mice fed with HFDs for different periods (16 to 24 weeks) had increased body weight, body fat percentage, and fasting blood glucose level, confirming the metabolic effects of this type of diet. In addition, changes in immune patterns were found with the enhanced accumulation of immune cells in AT, increased serum cytokines levels, and an enlargement of adipocyte size [95]. The influx of macrophages and effector T cells in AT was also observed during human AT inflammation [96].

Likewise, in the context of CD, the infiltration of macrophages in AT was presented in both early and late infection in a murine model of diet-induced obesity. In this model, a medium-fat diet (MFD, 14%) was administered in association with fructose in water (5%) and a single dose of streptozotocin [27]. In infected mice fed with HFD, the number of inflammatory cells in AT and heart at 30 dpi is greater than that observed in *T. cruzi*-infected mice fed with a standard diet (SD, 10% fat) [17,73].

**Figure 3:**
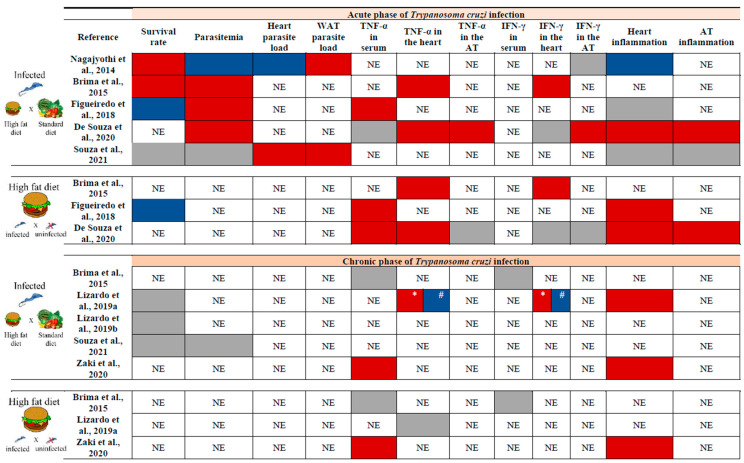
Impact of a high-fat diet on some variables analyzed in the acute and/or chronic phases of *T. cruzi* infection according to key studies. The upper section presents the results of experiments conducted during the acute phase of *T. cruzi* infection, while the bottom section corresponds to the chronic phase. In each section, the top panel compares infected animals fed with a high-fat diet (HFD) to those fed with a standard diet (SD), and the bottom panel compares infected and uninfected mice fed an HFD. The red box indicates an increase in the variable, the blue box indicates a decrease, and the gray box indicates no change. It is important to note that differences in results may be attributed to variations in experimental protocols. All studies were performed in murine models [17,63,70,71,72,73,74,75]. WAT: white adipose tissue. TNF-α: tumor necrosis factor-alpha. IFN-γ: interferon-gamma. AT: adipose tissue. NE: Not evaluated. *: Increase in 120 dpi. #: Decrease in 160 dpi.

The consumption of MFD resulted in both AT and systemic inflammatory response. Circulating levels of IL-6 were higher in acute and chronic phases compared to a group submitted to the MFD but not infected, while TNF-α showed a significant increase exclusively during the early infection [27]. In a model in which mice started HFD 8 weeks before infection, plasma TNF-α levels [73], as well as its concentrations in the heart [70,73], were elevated in the acute phase of infection, while the measurements in AT were similar between acutely infected and uninfected mice on HFD [73]. In the same way, the production of interferon-gamma (IFN-γ) in AT tissue was equivalent in groups submitted to HFD infected or not [73]. When comparing acutely *T. cruzi*-infected animals fed an SD to the HFD, divergent results concerning the levels of IFN-γ in cardiac and AT could be observed in different studies (Figure 3).

The expression of leptin, AD, and PPAR-γ, markers associated with lipogenesis and adipogenesis, were lower in the WAT of mice fed an SD compared to those fed an HFD during acute infection, whereas these adipokines were elevated in the heart, particularly in mice on the HFD [17]. In the chronic phase, serum leptin levels were reduced in uninfected MFD mice compared to their infected counterparts at 12- and 24 weeks post-infection. Conversely, AD levels were decreased in MFD-infected mice relative to uninfected ones in the same period [27].

There was a discrepancy regarding mortality and parasitemia during the acute phase. When comparing mice on HFD to those on SD, the survival rate was reported as higher [17,70], lower [71], or similar between the groups [74]. These differences may perhaps be attributed to the *T. cruzi* strain used in the studies (Brazil strain for [17,70]; VL-10 strain for [71]; Colombian strain for [74]).

Similarly, *T. cruzi*-infected mice on HFD exhibited varying levels of parasitemia, which were decreased [17,75], enhanced [70,71,73], or similar to those on SD [74]. Regarding parasite load, heart tissue showed a lower parasite burden with HFD [17], while an increase was observed in WAT [17,74]. Although Souza et al. [74] did not observe an elevation in parasitemia in infected mice fed with HFD compared to SD, the parasitic load in both heart and AT increased at 30 dpi with this dietary pattern [74] (Figure 3). Once again, some of these differences may be attributed to the experimental design, the key features of which are outlined in Table S1 (Supplementary Materials).

When associated with *T. cruzi* infection, HFD promotes adipogenesis [17] and cardiac lipidopathy, which may change the course of the acute phase. Indeed, Nagajyothi et al. [17] suggest that HFD has a protective effect against myocardial damage during the acute phase of *T. cruzi* infection, likely due to these mechanisms. Histology of the heart from infected HFD-fed mice displayed a reduction in fibrosis and inflammation compared to infected SD-fed mice. Additionally, infected mice on HFD had only a 20% reduction in fat compared to uninfected HFD-fed mice, whereas infected SD-fed mice showed significant lipolysis [17].

In the chronic phase of infection, a link was suggested among the consumption of HFD, inflammatory status in the AT, and the progression and severity of CD. During this stage of the disease, the presence of inflammatory infiltrate was higher in the heart [63,75], as well as serum levels of TNF-α in mice fed with HFD and infected with *T. cruzi* [75] (Figure 3).

On the other hand, the ablation of WAT in mice did not increase the number of parasites but led to a redistribution of *T. cruzi* in different tissues, resulting in a decreased parasitic load in the epididymal WAT. These mice also showed higher cardiac inflammatory infiltrate compared to infected wild-type mice. In other words, fat ablation may exacerbate the inflammatory response during *T. cruzi* infection, potentially intensifying cardiac damage and Chagas cardiomyopathy [75].

According to Lizardo et al. [63], mice exposed to an HFD and infected with *T. cruzi* are more susceptible to cardiac alterations in the late chronic phase (160 dpi). These alterations affected left and right ventricular function, accompanied by tissue damage characterized by heightened inflammation and hypertrophy. Furthermore, HFD exacerbates mitochondrial stress, which the authors suggest may contribute to the pathogenesis of CCC [63].

Altogether, these findings indicate that dietary lipid content influences the progression of CD by impacting immune responses, inflammation, and lipid metabolism. In fact, *T. cruzi* demonstrates a strong affinity for host lipoproteins and cholesterol, utilizing lipids and the low-density lipoprotein receptor (LDLr) for cell invasion [97].

It is essential to note that HFD consumption exhibits mixed effects on CD, depending on the disease stage, *T. cruzi*’s genetic background, the host’s immune response, and the overall metabolic status, including AT metabolism (Figure 3). This underscores the intricate nature of CD’s pathogenic process. It is important to emphasize that the lack of agreement regarding assay design may explain divergent results and, at times, lead to inconsistencies when comparing studies.

The literature review pointed out that a greater number of analyses were performed in the acute phase of disease, yielding both positive and negative results (Figure 3). In the chronic phase of infection, although a certain number of variables presented in Figure 3 do not change with the consumption of HFD, the results of the different cardiac analyses indicate that this diet is detrimental in the long term, contributing to the progression of CCC [63,75].

Thus, the studies conducted to date suggest that the consumption of an HFD can modulate the progression of CD by influencing immune responses, inflammatory processes, and lipid metabolism. The HFD induces notable alterations in AT, characterized by increased infiltration of inflammatory cells and modifications in adipokine expression. Additionally, this dietary pattern impacts parasite load and cardiac responses. While the effects of HFD during the acute phase of infection exhibit variability, in the chronic phase, it appears to exacerbate cardiac inflammation and mitochondrial dysfunction, thereby amplifying myocardial damage and contributing to the progression of CCC (Figure 3). Together, these results highlight the necessity for further investigation into the impact of diet on the course of *T. cruzi* infection.

## 6. Conclusions

**Figure 4:**
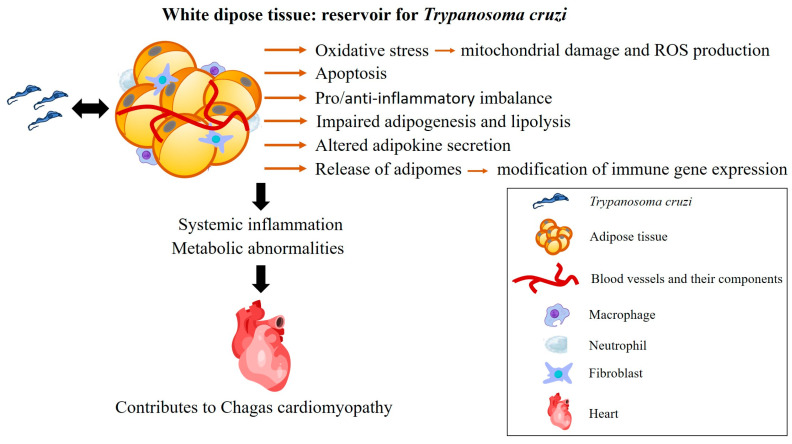
Consequences of the interaction between *T. cruzi* and white adipose tissue. In the acute phase of infection, adipose tissue is a target for *T. cruzi*. In the chronic phase, it serves as a reservoir for the parasite, contributing to the persistence of the infection. The inflammation triggered by *T. cruzi* persists in adipose tissue, leading to several effects, including oxidative stress, adipocyte apoptosis, an imbalance in pro- and anti-inflammatory factors, as well as impaired adipogenesis and lipolysis. These effects are influenced by the parasite’s genetics and the host’s immune response. Together, these factors contribute to the development of chronic Chagas cardiomyopathy. ROS: reactive oxygen species. Heart (https://beta.scidraw.io/drawing/277) and macrophage (https://beta.scidraw.io/drawing/221) were obtained from https://scidraw.io/. Accessed on 19 February 2025.

Despite the evidence, limited attention has been given to the clinical importance of AT in the pathological progression of CD. This review underscores the potential role of AT in the pathogenesis of CD, as it is a complex organ composed of several cell types with immunological and hormonal functions through the release of numerous adipokines and cytokines involved, among others, in inflammatory pathways. Moreover, *T. cruzi* persists within adipocytes, contributing to systemic inflammation and metabolic abnormalities that can exacerbate CD severity (Figure 4). The interplay between cardiac tissue and AT warrants further investigation, as both are affected by *T. cruzi* infection, and cardiac fat appears to play a role in the regulation of metabolic processes and myocardial inflammation. Furthermore, the influence of HFD on CD progression also reveals complexities. While HFD may offer protection against acute cardiac damage, it could potentially exacerbate inflammation and myocardial alterations in the chronic phase, thereby worsening CCC. The influence of normal and dysfunctional AT on the severity of CD remains unclear and should be further investigated, especially the role of myocardial fat. Elucidating the role of AT in CD pathogenesis may allow advances in treatment strategies for the disease.

## Supplementary Materials

The following supporting information can be downloaded at https://www.mdpi.com/article/10.3390/pathogens14040339/s1, Table S1: Key characteristics of the main articles on the impact of high-fat diet on Chagas disease.

## Abbreviations

The following abbreviations are used in this manuscript:

CD	Chagas disease
AT	adipose tissue
HFD	high-fat diet
qPCR	quantitative PCR
kDNA	kinetoplast DNA
CCC	chronic Chagas cardiomyopathy
WAT	white adipose tissue
BAT	brown adipose tissue
miRNAs	microRNAs
PAT	pink adipose tissue
PBMCs	peripheral blood mononuclear cells
IL-6	interleukin-6
TNF-α	tumor necrosis factor-alpha
PPAR-γ	peroxisome proliferator-activated receptor gamma
AD	adiponectin
ATF-7	activating transcription factor-7
BMI	body mass index
dpi	days post-infection
MFD	medium-fat diet
IFN-γ	interferon-gamma
SD	standard diet
LDLr	low-density lipoprotein receptor
