# Supplementary material for: Adipose Tissue in Chagas Disease: A Neglected Component of Pathogenesis
Source: Pathogens. 2025 Mar 31;14(4):339. doi: 10.3390/pathogens14040339 (PMC12030347; doi:10.3390/pathogens14040339)
Supplement: Supplementary file 1 [file pathogens-14-00339-s001.zip › pathogens-3429657-supplementary.pdf]

Table S1. Key characteristics of the main articles on the impact of high-fat diet on Chagas disease.

Ref*	Mouse strain	Mouse age$	*T. cruzi* strain	Inoculum#	% fat diet@	Diet management	Euthanasia	Biological sample	Main variables analyzed
Nagajyothi et al., 2014	Male CD-1	8-10	Brazil strain	$5x10^4$	60%	Start at the day of infection until 35 dpi	35 dpi	Blood, heart and epididymal AT	Survival rate; lipid profile; parasite load; expression of adipogenic genes and genes involved in lipid metabolism; heart histopathology; metabolic state; cardiac function
Brima et al., 2015	CD-1	5	Brazil strain	$5x10^4$	60%	Start at 8 weeks before infection until 35 and 70 dpi.	35 and 70 dpi	Blood, liver, heart and WAT	Survival rate; fasting blood glucose; oral glucose tolerance; parasitemia; body composition; heart cytokines; At 70 dpi: plasma insulin and leptin; blood cytokines
Figueiredo et al., 2018	Male C57BL/6	3	VL-10 strain	$5x10^3$	60%	Start at 8 weeks before infection until 30 dpi	30 dpi	Blood, liver, and heart	Parasitemia; body composition; glucose and insulin tolerance; lipid profile; heart MMP-2 activity; plasma cytokines; heart and liver histopathology
De Souza et al., 2020	Female C57BL/6	3	VL-10 strain	$5x10^3$	60%	Start at 8 weeks before infection until 30 dpi	90 dpi	Blood, heart, liver and AT	Body mass; plasma lipid profile; CCL2 and plasma cytokines; hepatic lipids; heart and inguinal AT histopathology
Souza et al., 2021	Male C57BL/6	3	Colombian strain	50	60%	Start at 8 weeks before infection until 30 dpi	30 and 60 dpi	Blood, heart, liver, and epididymal AT	Survival rate and parasitemia (until 60 dpi); body composition; plasma biochemical variables; hepatic lipids; heart and AT parasite load; heart and AT CCL2 levels; redox status; heart and AT histopathology
Lizardo et al., 2019a	Male CD-1	6-8	Brazil strain	10^3	60%	Start at 35 dpi until 120 and 160 dpi	120 and 160 dpi	Blood, heart, and liver	Survital rate; cardiac morphology; expression of genes involved in lipid metabolism; oxidative stress and inflammatory signaling; heart histopathology; heart and liver cholesterol; heart RAGE levels
Lizardo et al., 2019b	Male CD-1	6-8	Brazil strain	$5x10^3$	60%	Start at 35 dpi until 150 dpi	150 dpi	Blood, liver, and heart	Survival rate; body weight; fasting blood glucose; oral glucose; serum metabolomic biomarkers; heart and liver cholesterol; heart morphology
Zaki et al., 2020	Swiss female	4	Berenice strain	$5x10^3$	20%	Start at the time of infection until 90 dpi	90 dpi	Blood, heart, WAT	Body weight; parasitemia; plasma cholesterol and glucose; heart and retroperitoneal AT histopathology

*Reference. $in weeks. #Via intraperitoneal. @A multitude of diets can be used in murine studies. The lack of standardization
RAGE: receptor for advanced glycation end products. dpi: days post-infection. AT: adipose tissue. WAT: white adipose tissue.
